# Bee Threat Elicits Alarm Call in African Elephants

**DOI:** 10.1371/journal.pone.0010346

**Published:** 2010-04-26

**Authors:** Lucy E. King, Joseph Soltis, Iain Douglas-Hamilton, Anne Savage, Fritz Vollrath

**Affiliations:** 1 Animal Behaviour Research Group, Department of Zoology, University of Oxford, Oxford, United Kingdom; 2 Education and Science, Disney's Animal Kingdom, Bay Lake, Florida, United States of America; 3 Save the Elephants, Nairobi, Kenya; University of Sussex, United Kingdom

## Abstract

Unlike the smaller and more vulnerable mammals, African elephants have relatively few predators that threaten their survival. The sound of disturbed African honeybees *Apis meliffera scutellata* causes African elephants *Loxodonta africana* to retreat and produce warning vocalizations that lead other elephants to join the flight. In our first experiment, audio playbacks of bee sounds induced elephants to retreat and elicited more head-shaking and dusting, reactive behaviors that may prevent bee stings, compared to white noise control playbacks. Most importantly, elephants produced distinctive “rumble” vocalizations in response to bee sounds. These rumbles exhibited an upward shift in the second formant location, which implies active vocal tract modulation, compared to rumbles made in response to white noise playbacks. In a second experiment, audio playbacks of these rumbles produced in response to bees elicited increased headshaking, and further and faster retreat behavior in other elephants, compared to control rumble playbacks with lower second formant frequencies. These responses to the bee rumble stimuli occurred in the absence of any bees or bee sounds. This suggests that these elephant rumbles may function as referential signals, in which a formant frequency shift alerts nearby elephants about an external threat, in this case, the threat of bees.

## Introduction

Mammalian calls can reflect the internal states of animals, such as fear, but also may refer to external objects or events, such as the presence of predators [1]. For example, arousing social contexts including social separations or encounters with strangers can result in calls of increased emotional intensity as observed in rhesus monkeys, *Macaca mulatta* [2], red fronted lemurs, *Eulemur rufifrons* [3], baboons, *Papio cynocephalus ursinus* [4], guinea pigs, *Cavia porcellus* [5], and tree shrews, *Tupaia belangeri* [6]. Typical acoustic responses to potentially threatening challenges include changes in tempo-related features (e.g. call rate and duration) and source features (e.g. increased and more variable frequency and amplitude). Filter features related to vocal tract modulations are less commonly associated with arousal, but have been observed in baboons [4].

In addition to expressing internal state, mammalian vocalizations are also known to refer to external objects or events (i.e., ‘referential signaling’ [1]). In many cases, mammalian alarm calls vary acoustically according to specific predator species or class of predator (e.g., aerial versus terrestrial). Playback experiments with suricates, *Suricata suricatta* [7], and vervet monkeys, *Cercopithecus aethiops* [1], show that listeners react to alarm calls as if they were in the presence of an actual predator. This suggests that the acoustic structure of alarm calls can be related to specific external events, which in turn can be acted upon in adaptive ways by listeners. The complexity and variation of the acoustic cues can be seen in examples taken from three species of *Cercopithecus*, in which vervet monkeys *C. aethiops* separate their alarm calls for leopards and eagles through the location of dominant frequencies [8], Campbell's monkeys *C. campbelli* separate them by call duration, fundamental frequency and dominant frequency location [9], while Diana monkeys *C. diana* separate them by call rate, duration, fundamental frequency and formant frequency location [10], [11], [12]. Animal alarm calls are not always predator specific, however. For example, yellow-bellied marmot, *Marmota flaviventris*, alarm calls are similar towards a range of predators but do increase in rate with level of perceived risk [13].

Unlike the smaller and more vulnerable mammals, African elephants have relatively few predators that threaten their survival in the wild. In Kenya's Amboseli National Park, however, defensive and retreat behavior in elephants was observed in the presence of Masaai tribesman [14], who have been known to kill elephants. African elephants react similarly to sound playbacks of unfamiliar conspecifics [15]. Little research has been conducted on elephant vocalizations in response to specific threats, although observations of elephants ‘roaring’ or ‘trumpeting’ in response to the presence of lions is well known [16]. More recently, research has demonstrated that African elephants actively avoid contact with African honey bees - with implications for the management of both species [17], [18]. First was the discovery that Kenyan elephants avoid feeding on trees with beehives [19]. Subsequently, a playback study demonstrated that elephants retreat when hearing the sounds of disturbed bees [20].

In order to investigate this apparent natural threat to elephants further, we recorded the vocalizations of elephants in response to playbacks of disturbed bee sounds, using an array of microphones capable of recording low frequency elephant calls. In a second playback experiment, we played the recorded “rumble” vocalizations to resting elephants in order to examine their potential function. We played natural and experimentally modified ‘bee-response’ calls, in order to isolate and explore the effect of a specific acoustic feature on the response of listeners, namely, the location of the second formant. Such formant location shifts are due to modulations of the vocal tract [21]. Thus we were able to explore how an acoustically distinctive elephant rumble produced in the presence of bees may function as an alarm call.

## Results

### Honeybee playbacks

**Figure 1:**
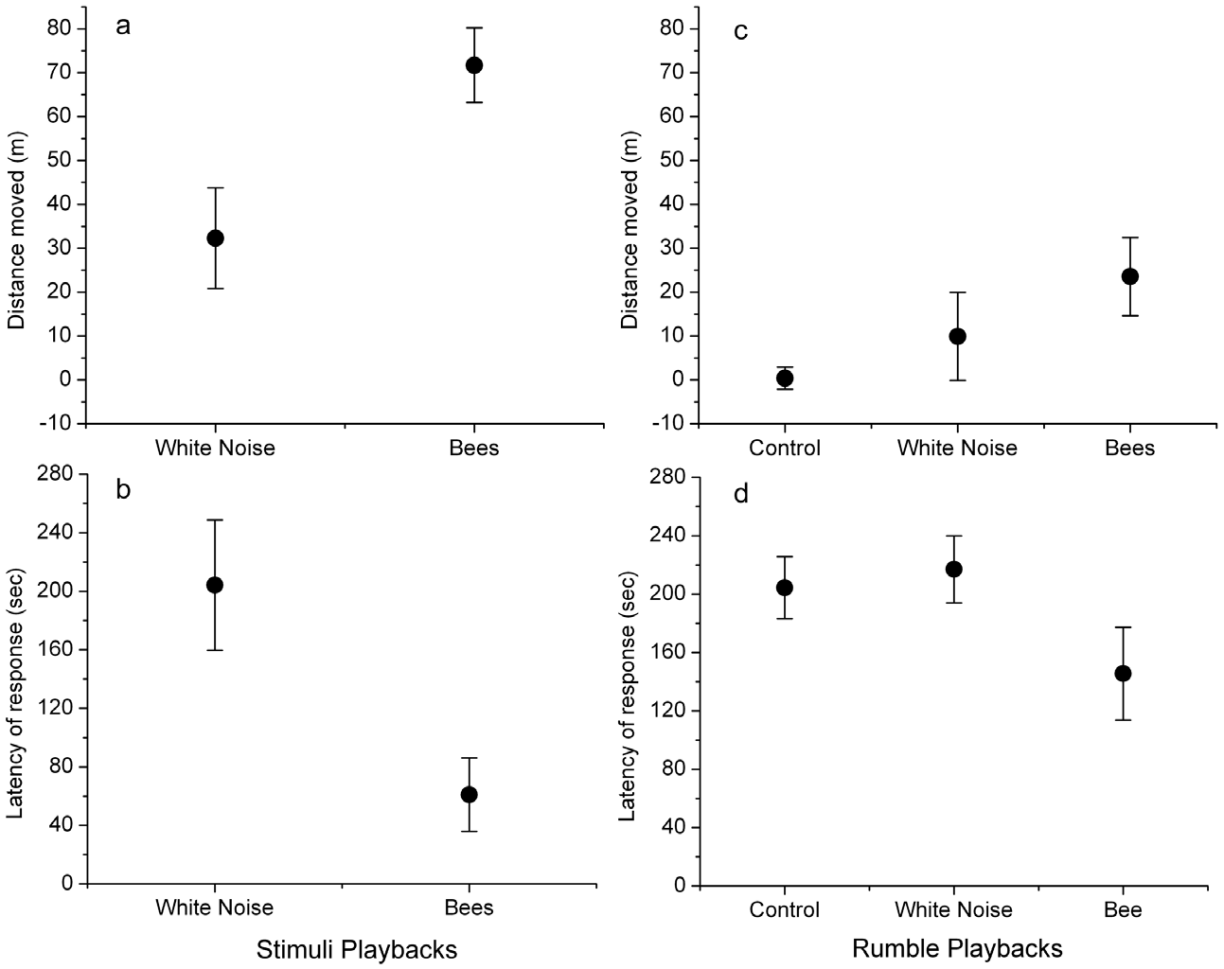
Distance moved and latency of response of elephants to sound and rumble playbacks. Mean (±1 SEM) of distance moved (a) and latency of response (b) of elephant families responding to bee sound (n = 15) and white noise (n = 13) playback trials. Elephants responding to bee sound playbacks moved on average over twice the distance of elephants responding to white noise playbacks (a) and were faster (b). For bee rumble playbacks (n = 10) elephant families moved away further (c) and faster (d) than elephant families responding to white noise or control rumble playbacks. Although rumble playbacks showed a more muted response than sound playback trials the directional pattern of behaviors were similar when comparing across experimental stimuli (a–d).

Confirming previous observations [20], elephants moved away in response to the playbacks of bee sounds. We performed 15 bee sound and 13 white noise playback trials to elephant families, consisting of a 2-min pre-stimulus phase, a 4-min stimulus phase (white noise or bee sounds), and a final 2-min post-stimulus phase. In 14 out of 15 bee trials (93%), families had moved away, compared to 6 of 13 white noise control trials (46%). Elephants moved away significantly further in response to bee sound playbacks (71.67 m ± s.e. 8.46) than to white noise playbacks (32.3 m ± s.e. 11.5; Mann-Whitney U test, n_1_ = 15, n_2_ = 13, U = 45, p = 0.012, Figure 1a). Additionally, using 360 seconds as a ceiling for families that did not move, elephants moved faster during bee sound playbacks (mean latency 61 sec ± s.e. 25.1; median: 25 seconds) than during white noise playbacks (mean latency 204 seconds ± s.e. 44.5; median: 207 seconds; Mann-Whitney U test, n_1_ = 15, n_2_ = 13, U = 56.5, p = 0.058, Figure 1b).

**Figure 2:**
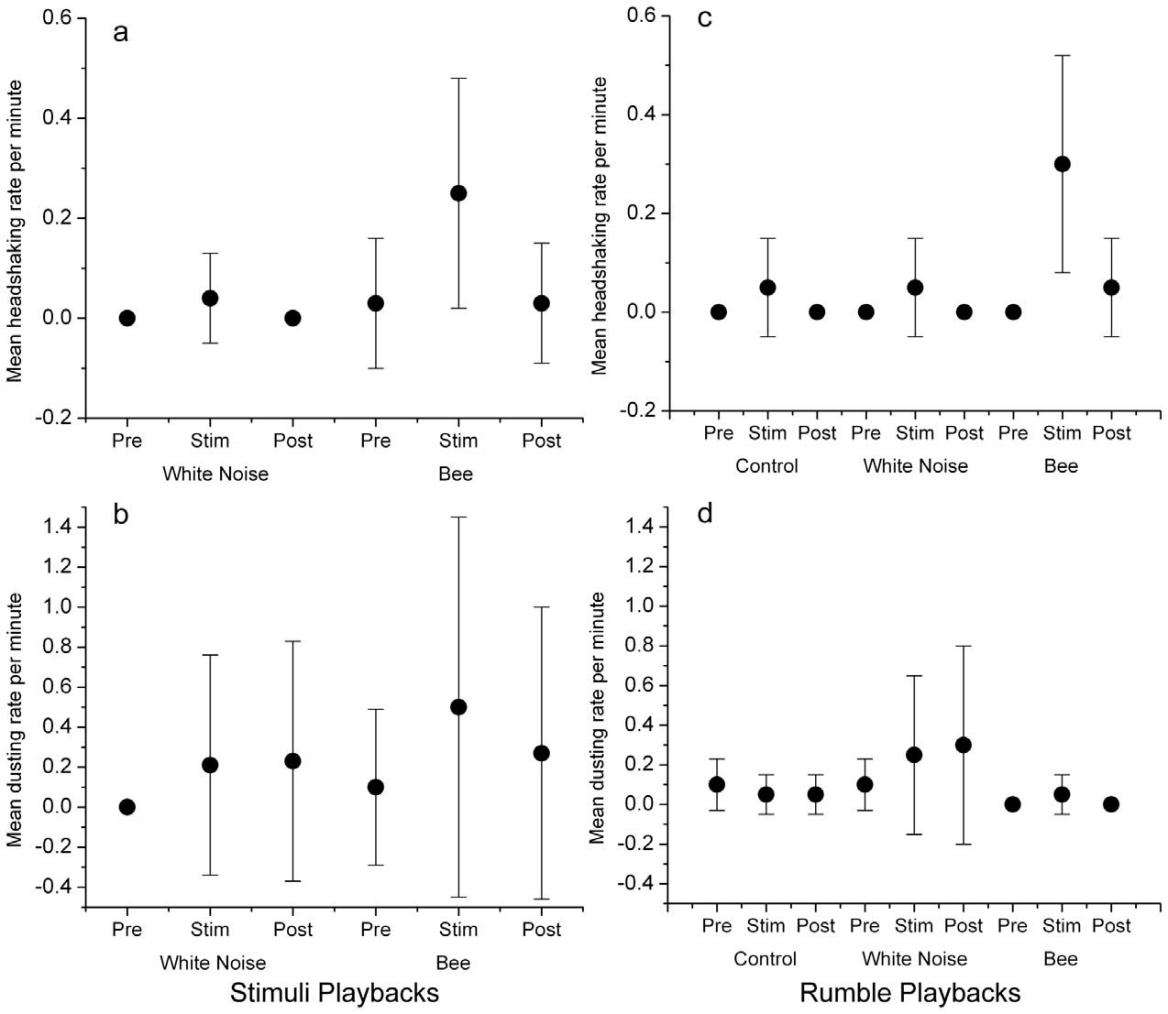
Headshaking and dusting behaviour of elephants responding to sound and rumble playbacks. Mean (±1 SEM) of headshaking (a) and dusting (b) rates per minute of elephant families responding to bee sound (n = 15) and white noise (n = 13) playback trials. Elephants responding to bee sound playbacks showed increased headshaking (a) and dusting (b) during the trials compared to those responding to white noise or control rumble playbacks. For bee rumble playbacks (n = 10) elephant families showed similar and significant patterns of increasing headshaking behavior (c) but dusting was random across trials (d).

Upon hearing bee sounds, elephants exhibited increased headshaking and dusting behavior during the 4-min stimulus phase of trials (Friedman's ANOVA, n = 15, headshaking: *F* = 6.4, *p* = 0.002; dusting: *F* = 5.7, *p* = 0.002; Figure 2a and 2b). When exposed to white noise, in contrast, headshaking and dusting were less frequent and rates did not differ across phases of the playback trials (Friedman's ANOVA, n = 13, headshaking: *F* = 0.55, *p* = 0.135; dusting: *F* = 1.19, *p* = 0.092; Figure 2a and 2b).

**Figure 3:**
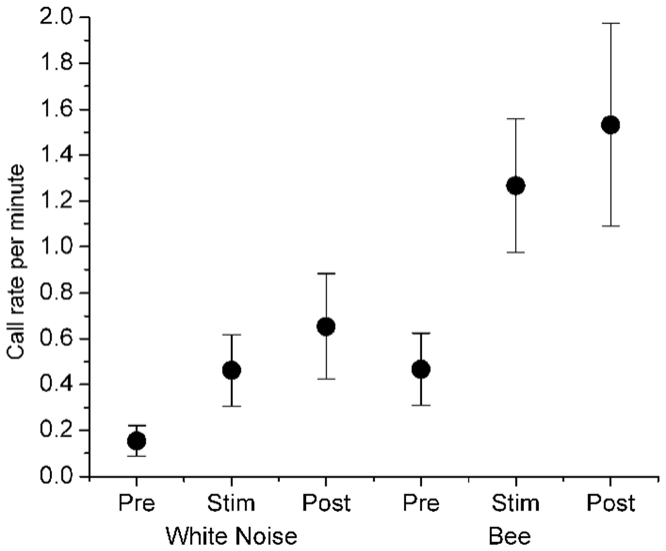
Call rates of elephants responding to sound and rumble playbacks. Mean call rates per minute (±1 SEM) recorded during the pre-stimulus, stimulus, and post-stimulus phases of bee (n = 15) and white noise (n = 13) playback trials. Elephants in bee playback trials responded to the stimuli with a significantly higher call rate in both the stimulus and post-stimuli phases compared to the pre-stimulus phase, but did not do so for white noise playback trials.

The total number of calls (rumbles, revs, screams, trumpets [22]) recorded from the triangular array was 217, and significantly higher for the bee sound playbacks (n = 15, calls = 160) than for white noise playbacks (n = 13, calls = 57; Kolmogorov-Smirnov two-sample test, χ^2^ = 10.03, *p* = 0.007) with low-frequency rumbles predominating (n = 199). During bee sound playback trials, call rates among non-infants (see Materials and Methods) was lowest during the pre-stimulus phase, increased during the bee stimulus phase, and remained high in the post-stimulus phase (Friedman's ANOVA, n = 15, *F* = 4.3, *p* = 0.046; Figure 3), but there was a muted response with no significant differences in call rates across trial phases for white noise playbacks (Friedman's ANOVA, n = 13, *F* = 3.04, *p* = 0.118). There were no significant differences between white noise and bee sound playback trials for family size, age composition within each trial family, microphone distances, temperature, time of day, altitude or air pressure (K-S two-sample tests, *p*>0.05).

### Acoustic properties of rumble response

We conducted acoustic measurements on rumbles occurring during the pre-stimulus phases of all trials (n = 13), during the stimulus and post-stimulus phases of bee sound trials (n = 20), and during stimulus and post-stimulus phases of white noise trials (n = 20; see Materials and Methods). Acoustic features measured were call duration, mean and range of the fundamental frequency, mean and range of call amplitude, and the first and second formant frequency locations [23]. Formants are enhanced frequency components of a call, produced by the resonating effects of the vocal tract filter, which enhance some frequencies (called resonant frequencies or formants) and diminish others [24]. MANOVA showed that the seven acoustic variables taken together differed across the three playback contexts (Wilks' Lambda = 0.484, *F*(14) = 2.745, *p* = 0.002). Univariate tests showed that the mean fundamental frequency (*F_o_*), the fundamental frequency range (max *F_o_*–min *F_o_*), and the second formant frequency location differed across playback contexts (ANOVA, *df* = 2, mean *F_o_*: *F* = 5.127, *p* = 0.009; *F_o_* range: *F* = 8.479, *p* = 0.001; second formant location: *F* = 5.817, *p* = 0.005).

**Figure 4:**
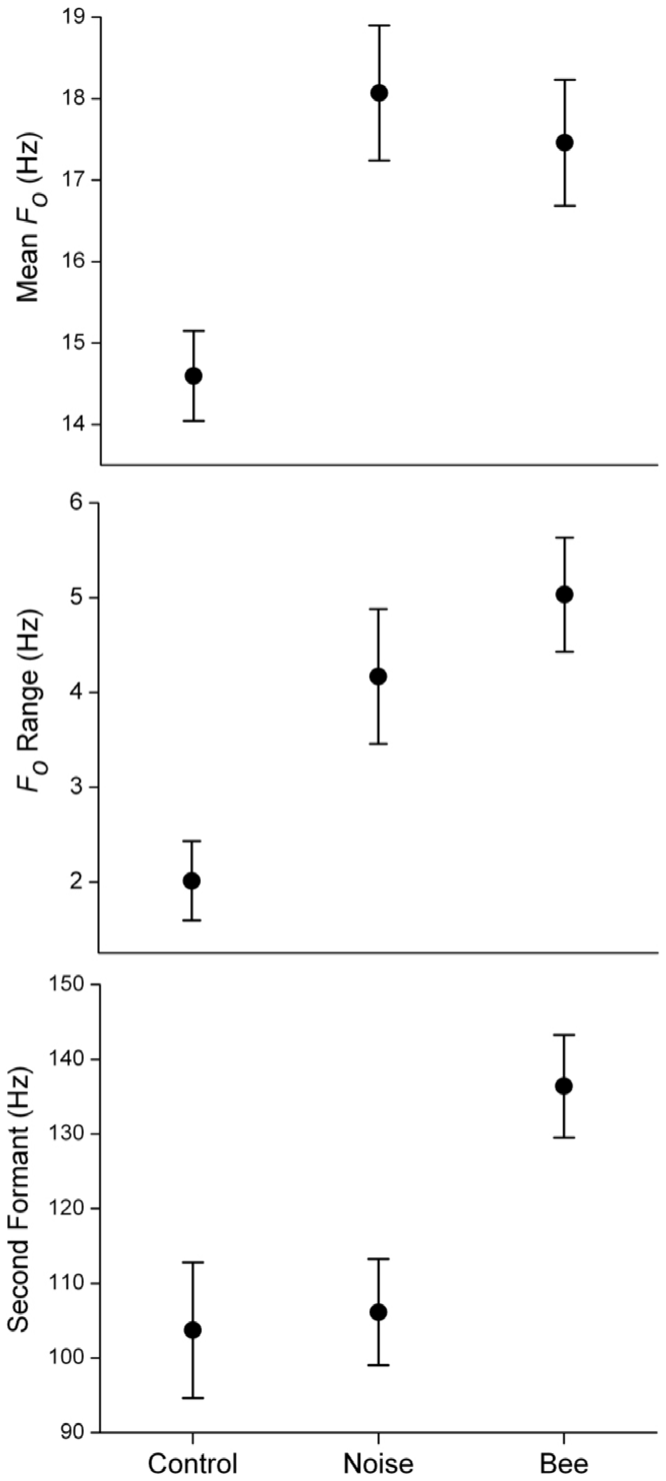
Acoustic features of rumbles emitted in response to sound playbacks. Mean (±1 SEM) for acoustic features across the three contexts (control  =  pre-stimulus phases of trials; noise  =  during stimulus or post-stimulus phases of white noise trials; bees  =  during stimulus or post-stimulus phases of bee trials). Results of pair-wise tests showed that bee and white noise rumbles were statistically different from controls for mean *F_o_* and *F_o_* range, and that bee rumbles were significantly different from white noise and control rumbles for second formant frequency location.

Tukey's Honestly Significant Difference pair-wise tests revealed that rumbles produced during white noise and bee sound trials both exhibited increased fundamental frequency and fundamental frequency range, compared to pre-stimulus control rumbles (*F_o_*: white noise vs. control *p* = 0.009, bee vs. control *p* = 0.036; *F_o_* range: white noise vs. control *p* = 0.020, bee vs. control *p*<0.001) (Figure 4). Additionally, rumbles produced during bee sound trials exhibited an upward shift in the second formant location, compared to both white noise (*p* = 0.013) and control rumbles (*p* = 0.018) (Figure 4). Observed acoustic changes were not attributable to body size or physical exertion, as no acoustic measure was significantly correlated with the age composition of the target family group or the distance moved away from playback stimuli (Pearson's correlations, *p*>0.05).

### Rumble Playbacks

**Figure 5:**
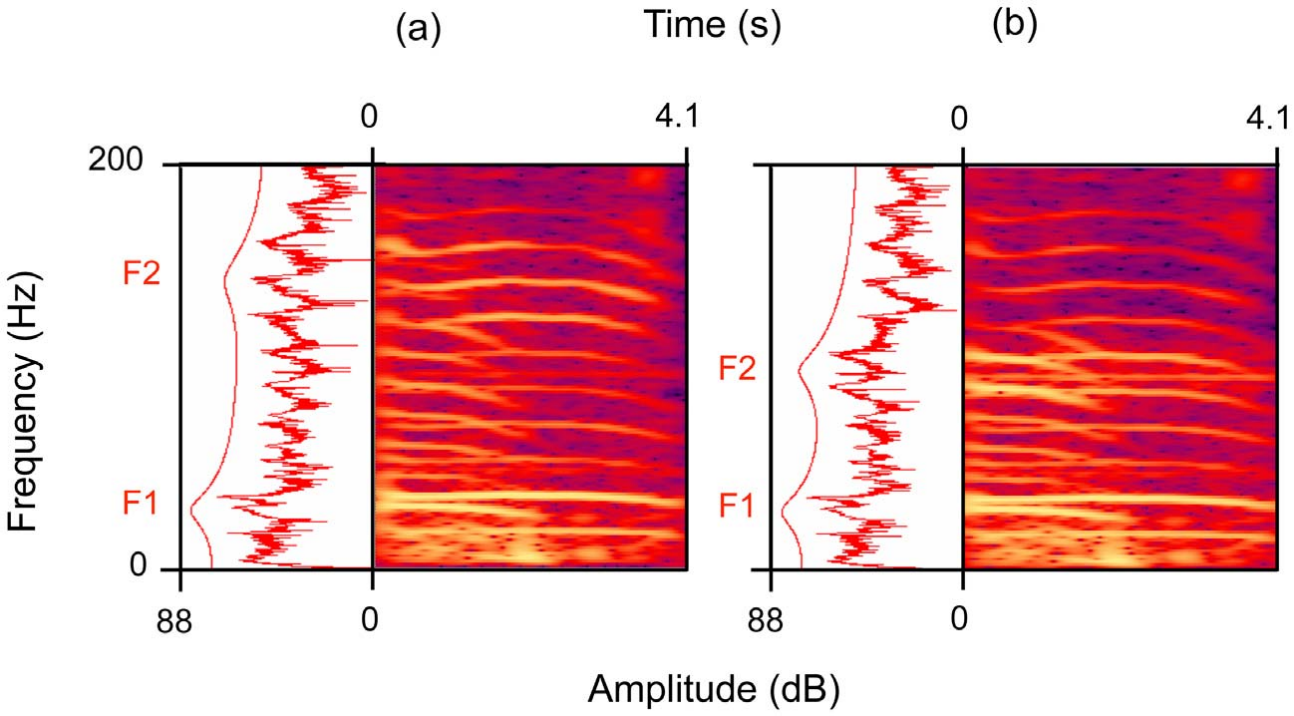
Spectrograms of elephant rumbles. (a) Unmodified African elephant rumble response to the bee playback stimulus. The Fourier frequency spectrum of the entire signal (PRAAT, version 4.6.18) with LPC smoothing showing two formants (F1, F2) and the spectrogram (44.1 kHz, Hanning window, 16384 bands; Adobe Audition, version 1.5) are shown. (b) Same signal as (a) with the frequency location of the second formants (F2) artificially lowered to match those observed in responses to white noise playbacks (see Materials and Methods).

We conducted a second playback experiment to determine if rumbles produced in response to bees elicit different responses in listeners compared to rumbles produced in response to white noise. However, we could not identify individual callers, so any differences observed in listener response to ‘bee’ and ‘white noise’ rumble playbacks could be due to individual variation of callers, not due to differences in the two classes of rumble (for details see Materials and Methods). We overcame this problem by experimentally manipulating rumbles produced in response to bees so that they resembled rumbles produced in response to white noise, namely, by lowering the second formant frequency location. We selected three bee response rumbles (Audio S1) that exhibited second formant frequencies that were typical of the class of bee rumbles as a whole (designated the ‘bee rumble’ stimulus). The ‘white noise rumble’ stimulus (Audio S2) consisted of the same three rumbles, but with the second formants experimentally lowered in frequency location to resemble rumbles produced in response to white noise playbacks (Figure 5; also see Materials and Methods). Thus, all features of the two stimuli remained identical, except the one feature that distinguished bee rumbles from white noise rumbles, the second formant location (compare Figures 4 and 5). As a further control, we selected three pre-stimulus rumbles from the same trial (‘control rumble’ stimulus), matched for duration and amplitude to those of the other rumble stimuli (Audio S3).

Rumble playback trials followed a similar protocol as the previous sound playback experiments, consisting of a 2-min pre-stimulus phase, followed by a 2-min stimulus phase (3 rumbles repeated 4 times), and a final 2-min post-stimulus phase. We performed 10 playbacks of each rumble stimulus (‘bee rumbles’, ‘white noise rumbles’, and ‘control rumbles’) in random order for a total of 30 playback trials. In 6 of the 10 bee rumble playback trials the elephant families moved away from the speaker (see online supplementary video, Video S1), compared to only 1 family moving away during 10 white noise rumble playbacks, and 2 families moving away during 10 control rumble playbacks (Table 1). It is possible that the order in which trials are presented can influence behavioral response, but there was no evidence for order effects in our trials. We were able to play more than one stimulus type to 11 families (Table 1), but there was no difference in distance moved when comparing the first and last playback trials (Wilcoxon Matched Pairs Test, n = 11, *p* = 0.969).

**Table 1:** Known elephant families tested with different rumble playback stimuli.

*Elephant Families Trials N = 30*	*Mean distance moved (m)*		
	*Bee*	*White Noise*	*Control*
Winds 2	60	0	12
Maya Churchill	80	−1	10
Winds 3	30	0	
Storms 2	0	0	
Spice Girls	8.6	0	
Butterflies	35	0	
Virtues: Hope	0		0
Virtues: Generosity	22		0
Artists 1	0		−18
Virtues	0		
Native Americans		100	
Winds 1		0	
First Ladies		0	0
Clouds		0	0
Artists 2			0
Rift Lakes: Baringo			0
Unknown Family			0

Distance moved was relative to the speaker during each playback trial. Minus sign indicates movement towards the speaker.

To detect differences in distanced moved from the speaker we conducted non-matched comparisons of the behavioural responses across ‘bee rumble’, modified ‘white noise rumble’, and ‘control rumble’ stimuli (Table 1). Elephant families exposed to the playback of bee rumbles moved away significantly further than elephants responding to either the white noise rumbles (Mann Whitney-U test, n = 10, U = 26, *p* = 0.041) or control rumbles (Mann Whitney-U test, n = 10, U = 24, *p* = 0.032), but distance moved was not different between white noise and control rumbles (Mann Whitney-U test, n = 10, U = 47, *p* = 1.0; Figure 1c).

Additionally elephants listening to bees moved faster than elephants responding to white noise (Mann Whitney-U test, n = 10, U = 26, *p* = 0.042; taking 240 seconds as the ceiling for elephants that did not move; Figure 1d) but a difference in latency between bee and control rumbles (Mann Whitney-U test, n = 10, U = 31.5, *p* = 0.132) and between white noise and control rumbles (Mann Whitney-U test, n = 10, U = 41.5, *p* = 0.582; were not significant.

Headshaking behavior increased significantly during the stimulus phase of the bee-rumble playbacks (Friedman's ANOVA, d.f. = 2, F = 3.15, p = 0.03) but no difference was observed across stimuli phases for families responding to white noise or control playbacks (Figure 2c). Headshaking behavior in response to bee rumble playbacks was remarkably similar to headshaking observed in direct response to bee sound playbacks (Figure 2a). Dusting was observed sporadically across all rumble trials but, unlike the response to bee sound playbacks (Figure 2b), did not increase in response to bee rumble playbacks (Figure 2d).

## Discussion

When exposed to the sounds of disturbed honeybees, African elephants exhibited behaviors that appear to function as defense against bees. Headshaking and dusting would knock bees away and fleeing from the area quickly would lower the risk of being stung. As elephants moved away from the sound source, they produced rumble vocalizations both during and after the bee sound stimulus. These rumbles may be simple expressions of emotional intensity [4], or they may function as contact calls that coordinate group movement [25], [26] or as alarm calls to more distant elephants [16], [25]. It is also possible that such calls are used in social facilitation i.e. teaching the inexperienced and more vulnerable young about a common and dangerous threat [15].

The acoustic characteristics of the rumbles we examined are consistent with both increased emotional intensity of callers and with signaling to conspecifics. For example, rumbles produced in response to bees and white noise both exhibited increased and more variable fundamental frequencies, two common acoustic features associated with increased emotional intensity in other mammals generally [4] and in African elephants specifically [23], [27]. However, rumbles produced in response to bees were further distinguished by an upward shift in the second formant location, which was not observed in white noise or pre-stimulus control rumbles, and has not been observed, to our knowledge, in other emotionally arousing contexts in elephants [23]. Such formant characteristics are controlled by the physical properties of the super-laryngeal vocal tract filter, which enhances resonance, or formant, frequencies. In humans, modulations of the vocal tract filter (e.g., lip rounding and tongue position) are responsible for the production of different vowels, which convey semantic information [24]. Our results suggest that such vocal tract manipulations in elephants may function in a similar way.

When rumbles produced in response to bees (with high second formant locations) were played to other elephant families, subjects were more likely to move further away from the sound source, and showed increased headshaking compared to reactions to the same rumbles with second formants artificially lowered to resemble ‘white noise’ rumbles, and to pre-stimulus control rumbles. Since the ‘bee rumbles’ and ‘white noise rumbles’ differed only in the location of the second formant, this provides evidence that vocal tract modulation alters the formant characteristics of their rumbles when in retreat from this threat, and that rumbles exhibiting such a formant frequency shift can function as a referential signal that warns other elephants about the presence of an external threat from the environment, in this case, the threat of bees.

While we cannot conclude with certainty that this alarm call is specific for bees (more experiments are underway to compare responses to other threats), the similar behavior patterns revealed in response to bee sound and to bee rumble playbacks (i.e., response speed, distance moved, and headshaking) make these calls good candidates for such specificity. Indeed, as elephants and bees have been interacting for millennia in the African savannah, selection pressure may have led to the evolution of an ability to communicate about such an ubiquitous threat, particularly in the light of the fact that other elephant vocalizations are situation specific [28], [29]. At the very least, rumbles with upwardly shifted second formant locations may function as general alarm calls, since other elephant families retreat far from the area when exposed to such rumbles in the absence of bees or other external threats. Dusting behavior increased in the presence of bee sounds, but did not increase during playbacks of ‘bee rumbles’, so more work is needed to reveal whether or not elephants might be trying to knock the insects out of the air with such behavior. Understanding how elephants react to and communicate about the presence of bees will not only advance our understanding of elephant behavior and vocal communication, but also our understanding of the potential deterrent effects of beehives on crop-raiding elephants [18].

## Materials and Methods

### Honeybee playbacks

We played the sounds of disturbed honeybees (n = 15) and white noise controls (n = 13) to elephant families containing known individuals resting under trees in the Samburu and Buffalo Springs National Reserves, Kenya [30], [31]. Following previously published protocols [20], we performed the playbacks from a camouflaged speaker (8–18 m from the nearest subject) in the dry season of February-March 2008. In addition, three audio-recording units were deployed in an array surrounding target families to capture their vocal response (44.1 kHz sample rate). Two units (Marantz PMD670 recorder; Earthworks QTC1 microphone, 4–40,000 Hz ±1 dB) were deployed from the vehicle window in duffle bags (15–70 m from nearest subject), and one unit (Marantz PMD671; Earthworks QTC50, 3–50,000 Hz ±3 dB) and a video recorder were deployed on the research vehicle roof (15–40 m from nearest subject).

After set-up, a two minute pre-stimulus control phase began, followed by a 4-min stimulus phase (bee sounds or white noise), and a final 2-min post-stimulus phase. After each trial, the distance that the elephants traveled away from the sound source was recorded (0–100 m [20]). Video of each trial was used to score other behaviors and group composition based on body size (age classes: 0–2 yrs, 3–14 yrs, >14 yrs). A minimum gap of 5 days was allocated before the same family was tested with the alternate sound. Every attempt was made to play both bees and white noise to the same family, randomly assigned, but some elephants left the reserve and were not see after the first trail.

The triangular array of three microphones surrounding the elephants allowed for the identification of vocalizations produced by the target family, by comparing relative amplitudes of calls on the three microphones. Identification of individual callers within families was not possible however. The number of calls (rumbles, revs, screams, trumpets [22]) recorded was 217 (n = 160 during bee playbacks; n = 57 during white noise playbacks). Low-frequency rumbles predominated (n = 199). Field observations suggested that infants vocalized at random across playback trials, so infant vocalizations (0–2 yrs) were removed from the data set. We identified infant rumbles using data from African elephant infants of known age (0–3 yrs; n = 120 rumbles) at Disney's Animal Kingdom [32], in which infants aged 0–2 yrs produced rumbles with mean fundamental frequencies above 20 Hz and mean durations below 1.5 sec. Rumbles meeting both criteria (n = 17) were removed.

### Acoustic measurement of rumble response

Rumbles were cut from start to end using Adobe Audition (version 1.5) and acoustic measurement of calls was performed in PRAAT (version 4.5.18) [33] using automated routines. Elephant rumbles were down-sampled to a 400 Hz sample rate to analyze low frequencies. For each call, pitch floor and ceiling variables were adjusted to surround the observed fundamental frequency, replacing standard settings. From the fundamental frequency (*F*_0_) contour, mean *F*_0_ and *F*_0_ range (maximum *F*_0_–minimum *F*_0_) were computed. From the intensity contour, mean amplitude and amplitude range were computed. Calls were high-pass filtered (Hanning window, 10 Hz cut-off, 1 Hz smoothing) to remove background noise below the signal. A Fast Fourier frequency spectrum of the middle 0.5 s of the call was generated (bandwidth = 200 Hz), from which the first two formant frequency locations were extracted by LPC-smoothing without pre-emphasis. Duration was defined as the length of the sound file.

Signal to noise ratio was sufficient to make full measurements on 132 of the 199 rumbles (66%). After removing infant rumbles (n = 12), there remained 13 pre-stimulus ‘control’ rumbles, 35 ‘white noise’ rumbles and 72 ‘bee’ rumbles. We selected for analysis all 13 pre-stimulus control rumbles and a random 20 rumbles from the ‘noise’ and ‘bee’ categories. The 13 pre-stimulus control rumbles were derived from 7 different families across 9 separate trials. The 20 noise and bee stimulus rumbles were each derived from 9 different families across 9 separate trials.

### Rumble playbacks

We conducted a second playback experiment to determine if the class of rumbles produced in response to bees elicits different responses in listeners compared to the class of rumbles produced in response to white noise. When comparing calls of two general classes such as these, the calls are likely to vary within each class (due to inter and intra-individual variation) as well as between classes. Therefore, any difference in response by listeners to playback rumbles could be attributable to individual variation (or some other idiosyncratic attribute of the recordings), and not to between-class differences in call stimuli [34]. One way to overcome this problem is to choose many different calls from each class for playbacks, so that such differences “average out”. However, in our case, we do not know the individual identity of callers, so that any observed difference in listener response could still be attributable to differences in the identity of specific callers, not to differences between ‘bee’ and ‘white noise’ rumbles.

Another means to overcome this problem, and the one we adopted here, is to experimentally manipulate calls so that the only acoustic difference between playback stimuli is the acoustic property of interest [34]. The only acoustic difference between rumbles produced in response to bee sounds and those produced in response to white noise was the location of the second formant frequency, so we manipulated this feature. Rumbles used for playbacks were extracted from audio recordings of a single bee sound playback trial on a mid-ranking, resident family [31]. ‘Bee rumbles’ consisted of three post-stimulus phase rumbles (duration = 9.4 sec) and exhibited second formant frequency locations typical of the ‘bee rumble’ class as a whole (Figure 4). To experimentally produce ‘white noise rumbles’, the second formants of the ‘bee rumbles’ were artificially lowered (Adobe Audition, version 1.5) to mirror the formant locations observed in rumbles produced during white noise playbacks (Figure 4). For one sequence of two rumbles, the frequencies associated with second formants (115–168 Hz) were reduced in amplitude (−10 dB), and lower frequencies (86–115 Hz) were amplified (+10 dB), shifting the second formant location from 132.3 to 104.5 Hz (Figure 5). For the third ‘bee rumble’, the 129–183 Hz band was reduced in amplitude (−10 dB), and the 78–123 Hz band was amplified (+10 dB), shifting the second formant location from 148.6 to 103.8 Hz.

In this way, we controlled for individual differences and the problem of ‘pseudo-replication’ [34]. This is because the unmodified ‘bee rumble’ stimulus exhibited high second formants that were representative of bee rumbles in general, and the experimentally modified ‘white noise rumble’ stimulus was identical in all respects (including individual identity), except that the formant locations were experimentally lowered to locations representative of the white noise rumbles in general (compare Figures 4 and 5). As a further control, three rumbles were isolated from the pre-stimulus phase of the same trial (duration = 8.3 sec), designated ‘control rumbles’.

All three rumble stimuli were matched for amplitude and speaker distance during playbacks. First, all stimuli were low-pass filtered (Adobe Audition, version 1.5; Butterworth filter, 1000 Hz cut-off), and were played from an FBT MAXX 4A speaker (frequency response: 50–20,000 Hz). Re-recording of test rumbles at 1 m showed amplitude loss below 50 Hz but frequency components were reproduced down to 20 Hz. Mean amplitudes across rumble sequences played from the FBT MAXX 4A speaker were 96.7, 96.2 and 95.7 dB (at 1 m) for the ‘bee’, ‘white noise’ and ‘control’ rumble stimuli, respectively (CEM DT-8852 Sound level meter data logger, slow, C weighting, sampling rate: 0.5 sec). In the field, the camouflaged speaker system was deployed 40–50 m from target families. Mean speaker distance from the nearest subject was 42.4, 43.2 and 42.2 m for the ‘bee’, ‘white noise’ and ‘control’ rumble stimuli, respectively.

The rumble stimuli were played back in random order until each stimulus type was played 10 times (n = 30 trials) in February 2009, using the same methods described previously for bee and white noise playbacks. After set-up, a two minute pre-stimulus control phase began, followed by a 2-min stimulus phase during which three rumbles were repeated four times (either ‘bee’, ‘white noise’ or ‘control’ rumble stimuli), and a final 2-min post-stimulus phase. After each trial, the distance that the elephants traveled away from the sound source was recorded (0–100 m [20]). We attempted to play all three stimuli to the same family groups but were not able to do so in all instances. Distance moved from the speaker was estimated in the field. Where partial group movement was observed, the mean distance moved was recorded. Behavioral responses and group compositions were scored from video.

### Statistical analyses

Behaviour was compared across playback contexts using non-parametric tests (GenStat, version 11.1). MANOVA was used to analyze rumble structure across experimental contexts (SPSS, version 15.0). Type III sum of squares was employed to correct for imbalanced data [35]. We used Pearson's correlations to examine relationships between individual acoustic features and a) the distance elephants moved away from the stimulus and b) the age composition of the target family group (adults/adults + juveniles). Two tailed alpha was set at .05 for all tests.

### Ethics statement

This research on wild African elephants was reviewed from an animal welfare perspective by Disney's Animal Care and Welfare Committee, and was approved on December 12, 2007. Clearance for research was granted by the National Council of Science and Technology, Republic of Kenya (No. NCST/5/002/R/1189; 31 Dec 2006–31 Jan 2013).

## Supporting Information

Audio S1 Recording of Bee Rumble. These three “bee rumbles” were recorded from an elephant family responding to bee stimuli and were used in the rumble playback experiments.
(0.40 MB WAV)

Audio S2 White Noise Rumble. These three “white noise rumbles” were recorded from an elephant family responding to bee stimuli where the second formants were experimentally lowered in frequency location to resemble rumbles produced in response to white noise playbacks.
(0.36 MB WAV)

Audio S3 Control Rumbles. These three “control rumbles” were recorded pre-stimulus from the same elephant family and were matched for duration and amplitude to the other rumble playbacks.
(0.57 MB WAV)

Video S4 Butterfly Family Response to Bee Rumble Playback. This video shows a typical response by elephants to the bee rumble playback. Here the Butterfly Family are resting under a tree when the rumble is heard to the right of the picture coming from the hidden wireless speaker. The response to move away is quick and the matriarch is seen headshaking as she walks away (in the opposite direction to the speaker) with her family.
(3.61 MB MOV)

## References

[1] Seyfarth RM, Cheney DL (2003). Signalers and receivers in animal communication.. Annu Rev Psychol.

[2] Bayart F, Hayashi KT, Faull KF, Barchas JD, Levine S (1990). Influence of maternal proximity on behavioural and physiological responses to separation in infant rhesus monkeys.. Behav Neuro.

[3] Fichtel C, Hammerschmidt K (2002). Responses of redfronted lemurs to experimentally modified alarm calls: evidence for urgency-based changes in call structure.. Ethology.

[4] Rendall D (2003). Acoustic correlates of caller identity and affect intensity in the vowel-like grunt vocalizations of baboons. J Acoust Soc.. Am.

[5] Monticelli PF, Tokumaru RS, Ades C (2004). Isolation induced changes in guinea pig *Cavia porcellus* pup distress whistles.. Ann Braz Acad Sci.

[6] Schehka S, Esser KH, Zimmerman E (2007). Acoustical expression of arousal in conflict situations in tree shrews.. J Comp Physiol A.

[7] Manser MB (2001). The acoustic structure of suricates' alarm calls varies with predator type and the level of response urgency.. Proc R Soc Lond B.

[8] Seyfarth RM, Cheney DL, Marler P (1980). Vervet monkey alarm calls: semantic communication in a free-ranging primate.. Anim Behav.

[9] Zuberbuhler K (2001). Predator-specific alarm calls in Campbell's monkeys, *Cercopithecus campbelli*.. Behav Ecol Sociobiol.

[10] Zuberbuhler K, Noe R, Seyfarth RM (1997). Diana monkey long-distance calls: messages for conspecifics and predators.. Anim Behav.

[11] Zuberbuhler K (2000). Referential labelling in Diana monkeys.. Anim Behav.

[12] Riede T, Zuberbuhler K (2003). The relationship between acoustic structure and semantic information in Diana monkey alarm vocalizations.. J Acoust Soc Am.

[13] Blumstein DT, Armitage KB (1997). Alarm calling in yellow-bellied marmots: The meaning of situationally variable alarm calls.. Anim Behav.

[14] Bates L, Sayialel K, Njiraini N, Moss C, Poole J (2007). Elephants Classify Human Ethnic Groups by Odor and Garment Color.. Curr Biol.

[15] McComb K, Moss C, Durant S, Baker L, Sayialel S (2001). Matriarchs as repositories of social knowledge in African elephants.. Science.

[16] Langbauer WR (2000). Elephant communication.. Zoo Biol.

[17] Hoare RE (2000). African elephants and humans in conflict: the outlook for coexistence.. Oryx.

[18] King LE, Lawrence A, Douglas-Hamilton I, Vollrath F (2009). Beehive fence deters crop-raiding elephants.. Afr J Ecol.

[19] Vollrath F, Douglas-Hamilton I (2002). African bees to control African elephants.. Naturwiss.

[20] King LE, Douglas-Hamilton I, Vollrath F (2007). African elephants run from the sound of disturbed bees.. Curr Biol.

[21] Soltis J (in press). Vocal communication in African elephants (Loxodonta africana).. Zoo Biol.

[22] Leong KM, Ortolani A, Burks KD, Mellen JD, Savage A (2002). Quantifying acoustic and temporal characteristics of vocalizations for a group of captive African elephants Loxodonta *africana*.. Bioacoustics.

[23] Soltis J, Leighty KA, Wesolek CM, Savage A (2009). The expression of affect in African elephant *Loxodonta africana* rumble vocalizations.. J Comp Psychol.

[24] Titze IR (1994). Principles of Voice Production..

[25] Poole JH, Payne K, Langbauer WR, Moss CJ (1988). The social contexts of some very low frequency calls of African elephants.. Behav Ecol Sociobiol.

[26] Leighty KA, Soltis J, Wesolek CM, Savage A (2008). Rumble vocalizations mediate interpartner distance in African elephants *Loxodonta africana*.. Anim Behav.

[27] Wood JD, McCowan B, Langbauer WR, Viljoen JJ, Hart LA (2005). Classification of African elephant *Loxodonta africana* rumbles using acoustic parameters and cluster analysis.. Bioacoustics.

[28] McComb K, Reby D, Moss C (in press). Vocal communication and social knowledge in African Elephants..

[29] McComb K, Reby D, Baker L, Moss C, Sayialel S (2003). Long-distance communication of social identity in African elephants.. Anim Behav.

[30] Wittemyer G (2001). The elephant population of Samburu and Buffalo Springs National Reserves.. Afr J Ecol.

[31] Wittemyer G, Getz WM (2007). Hierarchical dominance structure and social organization in African Elephants, *Loxodonta africana*.. Anim Behav.

[32] Wesolek CM, Soltis J, Leighty KA, Savage A (2009). Infant African elephant rumble vocalizations vary according to social interactions with adult females.. Bioacoustics.

[33] Boersma P, Weenink D (2007). http://www.praat.org/.

[34] McGregor PK, Catchpole CK, Dabelsteen T, Falls JB, Fusani L (1992). Design of playback experiments: the Thornbridge Hall NATO ARW consensus..

[35] Shaw RG, Mitchell-Olds T (1993). ANOVA for unbalanced data: an overview.. Ecology.

